# Study of a Brazilian Family Presenting Non-syndromic hearing loss with mitochondrial inheritance

**DOI:** 10.1016/S1808-8694(15)31392-6

**Published:** 2015-10-17

**Authors:** Altair Cadrobbi Pupo, Sulene Pirana, Mauro Spinelli, Karina Lezirovitz, Regina C. Mingroni Netto, Lisandra S. Macedo

**Affiliations:** 1 PhD in Communication Disorders at UNIFESP, Head of Department at speech and hearing therapy clinic - PUC/SP; 2 PhD in Medicine at Universidade de São Paulo, Associate Professor at the Speech and Hearing Therapy School at PUC-SP; 3 PhD in Sciences - Communication Disorders at PUC-SP, Full Professor of Speech Therapy at PUC-SP; 4 MSc in Sciences at Universidade de São Paulo. PhD student at the Institute of Biosciences at USP, Biologist; 5 PhD in Sciences at Universidade de São Paulo, Professor at the Institute of Biosciences at USP; 6 Specialist Speech and Hearing Therapist at PUC-SP, Speech and Hearing Therapist at Hospital e Maternidade Alvorada. Pontifícia Universidade Católica de São Paulo and Universidade de São Paulo

**Keywords:** genetics, mitochondrial inheritance, hearing loss, nonsyndromic hereditary deafness

## Abstract

We hereby report on the audiological and genetic findings in individuals from a Brazilian family, with the following mitochondrial mutation A1555G in the 12SrRNA gene (MT-RNR-1). Nine individuals underwent speech, audiologic (tonal audiometry and logoaudiometry) and genetic evaluations. Eight individuals among the A1555G carriers were affected by hearing impairment and one person had normal hearing thresholds till the end of the present study. The audiologic evaluation results indicated normal hearing thresholds all the way to bilateral profound hearing loss with post-lingual onset and variable configuration. Two affected siblings presented progressive hearing loss. It was impossible to precise the time of hearing loss onset; however, the impairment was present in both children and adults. The genetic study revealed the A1555G mitochondrial mutation

## INTRODUCTION

It is estimated that 16% of the hearing loss cases in Brazil have confirmed genetic origins, while 70% of the patients have non-syndromic hearing loss. Recessive autosomal inheritance accounts for 80% of the non-syndromic inherited hearing loss cases; dominant autosomal patterns are seen in 10% to 20% of the cases; 2–3% of the cases are connected to chromosome X; and mitochondrial inheritance occurs in only 1% of the patients^1^. Non-syndromic sensorineural hearing loss is a challenging condition for physicians, audiologists, and geneticists. Etiologic diagnosis is hard to reach, and the condition is not accompanied by physical traits indicative of genetic inheritance. A high degree of clinical suspicion and comprehensive information on the patient’s family end up being required to properly diagnose and approach the condition.

Mitochondrial DNA mutations and deletions have been identified in non-syndromic sensorineural hearing loss patients. The mutation most commonly associated with maternal inheritance is A1555G on gene 12S rRNA, a relatively frequent finding among Asians. This mutation is found in approximately 3% of the Japanese, 5.3% of Indonesian and 0.5–2.4% of the European patients with sensorineural hearing loss ^2^.

The pathogenesis of this mutation is still largely unknown, despite the various papers in the literature published on the matter^3^. In non-syndromic patients, mitochondrial sensorineural hearing loss is the only present symptom. Some authors have reported on the correlation between this condition and hypersensitivity to aminoglycosides^1^, ^4^. In the United States, this mutation has been found in 15% of the patients with aminoglycoside-induced hearing loss^5^.

Mutation A1555G has been described as the main cause of aminoglycoside-induced hearing loss^2^. The mitochondrial DNA strips of 62 members of nine families with aminoglycoside-induced hearing loss patients were analyzed, and mutation A1555G on gene 12S rRNA was found in 20 members of five families. The authors concluded that there is a correlation between audiovestibular alteration and genetic susceptibility to the aminoglycoside ototoxicity of 6.

A study on the audiological traits connected to mitochondrial DNA mutation in a Chinese family with 41 hearing loss patients found that the condition was associated with mitochondrial mutation A1555G on gene 12S rRNA. The patients had symmetric bilateral sensorineural hearing loss - often progressive - with wide variation on the time of onset. The authors also mentioned that environmental factors may have had a determining role in the clinical manifestation of the mutation^7^.

The same audiological aspects were verified in another study that enrolled 21 Japanese families with this mutation. The authors also found that there were individual variations in the characteristics of the hearing loss, correlation with permanent tinnitus, with and without history of taking aminoglycosides, and more severe hearing loss among patients who took the antibiotic drug^8^.

A similar study done in Calabria enrolled 55 members of 6 families with the mutation. They were affected by non-syndromic, generally symmetric hearing loss involving mainly high frequencies. The disease varied from mild to moderate, and progressed slowly. Subjects previously treated with ototoxic drugs had severe hearing loss, and one of them was a deaf-mute^9^.

In a Danish population, the prevalence of sensorineural dysacusis related to alterations in mitochondrial DNA and the identified clinical signs indicated that hearing loss caused by this mutation has a wide range of phenotypes in audiological traits such as hearing loss grade, manifestation, and time of onset^10^.

A study done in Brazil on the prevalence of A1555G mitochondrial mutation and other mutations on mitochondrial gene RNAtSer(UCN)(MT-RNR-1) enrolled 203 individuals with hearing loss and found mutation A1555G in 2% (4 families) of the patients. Other mutations were not found in this study^11^, ^12^.

From the above mentioned studies, the ones that looked into audiological tests shed light on information and issues yet unaddressed by Brazilian studies. They indicate, for instance, that by analyzing affected families we can gather a broader perspective on the audiological characteristics and their evolution, as well as possible environmental factors. This study aimed to describe the audiological and genetic findings pertaining to a Brazilian family with mutation A1555G on mitochondrial gene 12SrRNA.

## CLINICAL CASES

This study describes a family of subjects affected by non-syndromic hearing loss characterized by patterns of mitochondrial inheritance. One of the patients (III-8) was seen by a speech therapist and was suspected to have inherited hearing loss. His family members were then invited to be clinically assessed. Nine individuals were examined by ENT, speech therapy, and audiology experts (tone audiometry, logoaudiometry, and BAEP in some cases) and underwent genetic testing. Other family members came only to perform genetic studies or for the described clinical exams. This study describes and analyzes the audiological findings of only the nine individuals that underwent the tests mentioned above, i.e., genetic tests and ENT, speech therapy, and audiological examination. This study was approved by the Ethics Committee for Research with Human Beings at the Institute of Biomedical Sciences under permit 023/CEP.

Genetic studies included the analysis of DNA extracted from lymphocytes. PCR followed by DNA digestion with restriction enzyme Hae III were used to search for mitochondrial mutation A1555G, as described by Estivill et al. (1998)^13^.

The speech therapy and audiological exams were done at the ENT outpatient ward at Universidade de São Paulo (USP) and at the clinic under the Division for Education and Rehabilitation of Communication Disorders at Pontifícia Universidade Católica de São Paulo (DERDIC/PUC-SP). Genetic tests were conducted at the Center for Human Genome Studies of the Department of Genetics and Evolutional Biology at IBUSP.

**Figure 1:**
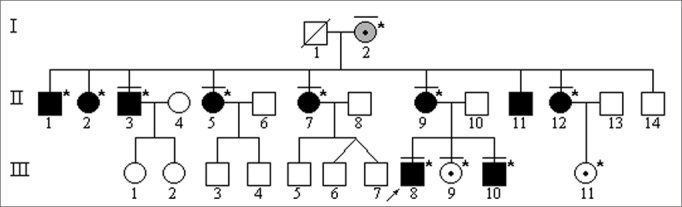
Family 14682 genealogical diagram (A). The arrow indicated purpose. Individuals marked in black have variable grades of hearing loss. Individuals marked with a dash above their symbols went through speech and hearing evaluation. Subjects marked with an asterisk were tested and have mitochondrial DNA mutation A1555G. (B) Polyacrylamide gel after impregnation with silver nitrate showing band pattern after PCR product digestion with restriction enzyme Hae III: normal control samples have 2 bands - 216pb and 123pb; family members carrying mutation A1555G have 3 bands - 216pb, 93pb, and 30pb.

All family members who underwent DNA analysis were found to have mutation A1555G on mitochondrial DNA gene RNAr 12S (Fig. 1).

**Figure 2:**
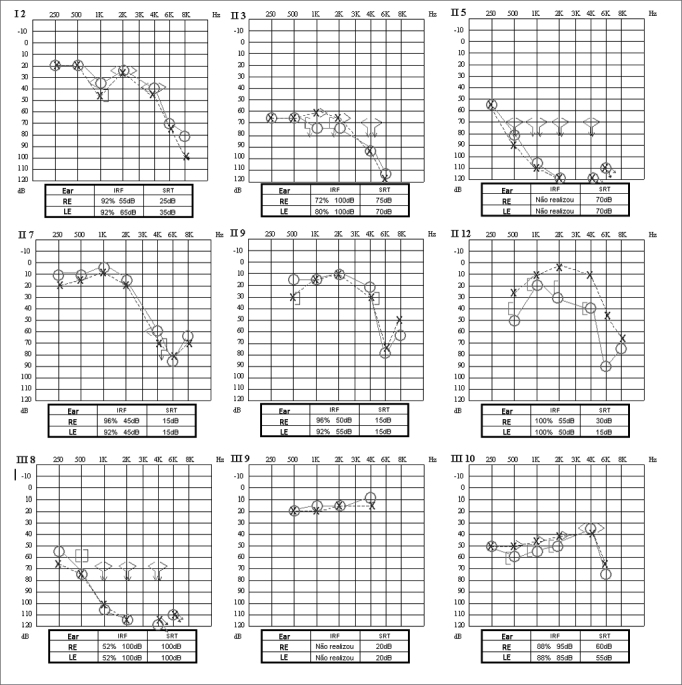
Audiometric profiles and logoaudiometry results of studied subjects.

One of the subjects (III-9) had no audiological symptoms, i.e., had normal hearing by the time of the tests. The other eight family members had different grades of bilateral, moderate to severe sensorineural hearing loss (Fig. 2). The data on all nine subjects, logoaudiometry results, and clinical information - mainly the data connected to the development of linguistic skills - indicated the presence of post-lingual hearing disorder. Symmetric hearing loss was more prevalent, as seen in other studies^7^, ^9^, except for subject II-12. Almost all hearing loss configurations -flat, low frequency, and high frequency - were seen. The high frequency type was nonetheless more prevalent than others, showing increased hearing involvement in high frequencies, as also seen in other studies^8^, ^9^. The other test results matched the clinical information.

**Figure 3:**
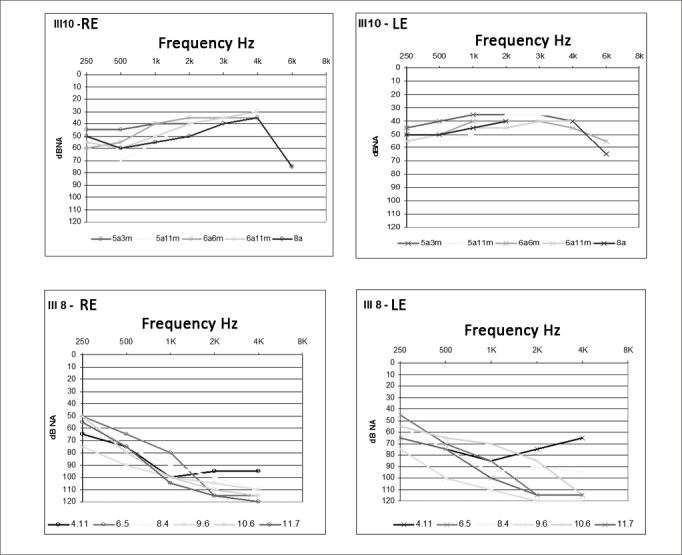
Hearing loss progression of subjects III-8 and III-10 - Colored legends indicate subject age at the time of the examination.

Audiological monitoring was done only for subjects III-8 and III-10 - two affected siblings - and their hearing loss was found to be worsening progressively. They had fluctuating, progressive hearing loss in both right and left ears (Fig. 3). Hearing loss progress findings are compatible with those seen in other studies^7^, ^8^, ^9^. The linguistic skills of the two affected siblings were not severely impaired, probably because their hearing loss was post-lingual, or because it was only a partial loss, this preserving their hearing within the speech frequency range. According to their parents, subject III-8 was born with good hearing and began to experience hearing loss at 4 years of age, while subject III-10 underwent audiological examination for the first time at the age of 5 years and 3 months for suspected hearing loss. The other subjects (I-2, II-3, II-5, II-7, II-9, and II-12) experienced hearing loss when they were adults.

Audiometric findings, considering the degree of hearing loss, indicated that clinical phenotypes varied quite substantially among the members of the studied family. The cases of hearing loss ranged from moderate to severe and were all bilateral and post-lingual. As mentioned above, there was no prevalent time of onset. Many of the subjects affected at adult age had tinnitus, and those treated for it had unsatisfactory outcomes^8^.

We could not connect the onset or worsening of hearing loss symptoms to the administration of aminoglycosides, as the subjects were unable to accurately report on the use of antibiotics.

Only one subject (II-3) with hearing loss had been previously exposed to occupational noise, one of the possible environmental factors leading to gene mutation expression^7^. Subject I-2 had mild hearing loss only at higher frequencies at 61 years of age, a condition possibly connected to presbycusis.

Subjects were informed of the importance of having their hearing status monitored and of the risks associated with taking aminoglycosides. Some started using hearing aids and greatly improved their oral communication skills and quality of life. The significant improvement in hearing tone and vocal thresholds confirmed the presence of cochlear involvement and stressed the absence of central hearing pathway involvement among patients with mitochondrial DNA mutation hearing loss.

## CLOSING REMARKS

Many studies have been produced to enhance the etiologic diagnosis of hearing loss. Technologic developments, new examination techniques, and genetic tests have allowed earlier and more accurate diagnosis, providing further clarification on the etiology of the condition.

Mitochondrial DNA mutations are an important cause of hearing loss. General practitioners should therefore be very careful when diagnosing the etiology of the condition, and use the valuable aid of genetic tests with that purpose. Clinical teams may thus assist in preventing the early onset and worsening of hearing loss cases, mitigating tinnitus prevalence, and advise patients on the risk of taking aminoglycosides and being exposed to noise for prolonged period of time.
